# The Potential Diagnostic and Predictive Role of HbA1c in Diabetic, Septic Patients: A Retrospective Single-Center Study

**DOI:** 10.1155/2022/8543232

**Published:** 2022-03-18

**Authors:** Imre Juhász, Janka Juhász, Hajnalka Lörincz, Ildikó Seres, Lilla Végh, Szilvia Ujfalusi, Mariann Harangi, Zoltán Szabó, György Paragh

**Affiliations:** ^1^Department of Emergency Medicine, Faculty of Medicine, University of Debrecen, Debrecen, Hungary; ^2^Doctoral School of Health Sciences, University of Debrecen, Debrecen, Hungary; ^3^Division of Metabolic Diseases, Department of Internal Medicine, Faculty of Medicine, University of Debrecen, Debrecen, Hungary

## Abstract

**Background:**

As diabetes mellitus is a major risk factor of sepsis, we aimed to evaluate the possible effects of diabetes mellitus and poor glycemic control on the diagnosis of sepsis.

**Methods:**

In our retrospective study, we included diabetic, septic patients—in whom the diagnosis of sepsis was based on the systemic inflammatory response syndrome (SIRS) criteria (*n* = 112, SIRS group)—who had HbA1c levels measured either in the previous 30 days (*n* = 39, SIRS 30 d subgroup) or within 24 hours after their emergency department admission (*n* = 73, SIRS 24 h subgroup). We later selected those patients from the SIRS group, whose sequential organ failure assessment (SOFA) score was ≥2 (*n* = 55, SOFA group), and these patients were also divided based on the time of HbA1c measurement (*n* = 21, SOFA 30 d subgroup and *n* = 34, SOFA 24 h subgroup). We analyzed the relationship between laboratory parameters, length of hospital stay, and HbA1c.

**Results:**

We found a significant positive correlation between glucose and HbA1c (*p* < 0.001, *p* < 0.001, respectively), significant negative correlations between white blood cell count (WBC) and glucose (*p*=0.01, *p*=0.02, respectively), WBC and HbA1c levels (*p*=0.001, *p*=0.02, respectively) in the SIRS 24 h and SOFA 24 h subgroups. Furthermore, there was a significant positive correlation between length of hospital stay and HbA1c in the SOFA 24 h subgroup (*p*=0.01). No significant correlations were found in the SIRS 30 d and SOFA 30 d subgroups.

**Conclusion:**

Based on our results, normal WBC with elevated HbA1c might be considered a positive SIRS criterium in diabetic, SIRS 24 h patients. Besides this potential diagnostic role, HbA1c might also be an additional prognostic biomarker in diabetic, SOFA 24 h patients.

## 1. Introduction

Sepsis is a potentially life-threatening condition. Its definition keeps on changing as we learn more and more about the underlying pathomechanism of the disease. Before 2016, the systemic inflammatory response syndrome (SIRS) criteria were used for the diagnosis of sepsis: if at least 2 out of 4 clinical findings were present in a patient with a likely infection, the diagnosis of sepsis was confirmed. The SIRS criteria include the following: tachycardia, hypothermia/fever, hyperventilation/hypocapnia, leukopenia/leukocytosis. The definition distinguished sepsis, severe sepsis, and septic shock [1–4]. In 2016, the definition of sepsis changed once again: the SIRS criteria, as well as the definition of severe sepsis, were no longer recommended. According to the new definition, sepsis is defined as a life-threatening organ dysfunction caused by a dysregulated host response to infection. The sequential organ failure assessment (SOFA) and the quick SOFA (qSOFA) scores have been introduced [4]. The diagnostic algorithm of sepsis has changed: in a patient with a likely infection, the use of the quick SOFA score is recommended (it consists of 3 components: systolic blood pressure ≤100 mmHg, altered mental status, and respiratory rate ≥22). According to the new recommendations, a positive qSOFA score (≥2 points) should prompt the calculation of the SOFA score to confirm the diagnosis of sepsis. If the qSOFA score is negative (<2 points), but sepsis is still likely, we should also calculate the SOFA score. In a patient with a negative qSOFA score (<2 points) and an unlikely infection, sepsis can be excluded. If a patient's SOFA score is ≥2, the diagnosis of sepsis is confirmed. The SOFA score consists of the following: platelet count, bilirubin, and creatinine levels, mean arterial pressure (MAP) or administration of vasoactive agents, altered mental status (based on the Glasgow Coma Scale), and PaO2/FiO2. Septic shock is a form of sepsis in which particularly profound circulatory, cellular, and metabolic abnormalities are associated with a greater risk of mortality than with sepsis alone. Patients with septic shock can be clinically identified by the vasopressor requirement to maintain a MAP of 65 mmHg or greater and serum lactate levels greater than 2 mmol/L in the absence of hypovolemia [4]. Lots of studies have been published since 2016 in which the SIRS, qSOFA, and SOFA criteria have been compared, and data are controversial [5]. Some results have shown inferior sensitivity of the qSOFA score compared to the previously favored SIRS criteria in the diagnosis of sepsis [6–9]. Partly due to this, the 2016 recommendations are not universally accepted, and many countries still favor the previous diagnostic criteria and therefore the SIRS criteria.

Sepsis is usually bacterial in origin (caused mainly by Gram-positive bacteria); however, viral and fungal causes could also be in the background [10]. Its global incidence is increasing, and it can be as high as 437/100000/year [11]. Major risk factors of sepsis include advanced age (≥65 years), previous hospitalization (especially in the previous 90 days, intensive care unit admission, nosocomial infections, community-acquired pneumonia), immunosuppression (e.g., neoplasms, renal failure, liver failure, AIDS, splenectomy), and genetic factors [10, 12–22]. Diabetes mellitus, a metabolic disorder that has become a global health burden partly due to its rising incidence, is another major risk factor for sepsis [23, 24]. Immune response is severely altered in diabetics: neutrophil chemotaxis, phagocytosis, intracellular bactericide activity, opsonization as well as cell-mediated immunity are all affected [25–28]. Therefore, infections are more common in diabetics compared to nondiabetic individuals. Poor glycemic control and hyperglycemia further increase the chance of infections in diabetics [25–30].

Hemoglobin in newly formed red blood cells is minimally glycated. The membrane of circulating red blood cells is permeable to glucose; therefore, it could be irreversibly attached to hemoglobin in a nonenzymatic way. HbA1c gives us information regarding mean blood glucose concentration over the lifespan of red blood cells (120 days), and its value correlates best with mean blood sugar levels over the previous 8–12 weeks. HbA1c is widely used nowadays to diagnose diabetes and monitor carbohydrate metabolism in diabetics [31–37]; however, its potential role in diabetic, septic patients has not yet been studied.

## 2. Materials and Methods

### 2.1. Study Participants

We collected all cases from the emergency department (ED) and later emergency clinic at the University of Debrecen, between 1 January 2017 and 31 December 2018 (27737 patients, 42766 cases). First, we selected patients who had their HbA1c measured in the study period (3743 patients), and later from these patients, we collected those diabetic, septic patients who had HbA1c levels measured either in the previous 30 days or within 24 hours after their ED admission. Sepsis was diagnosed based on the SIRS criteria. Patients with autoimmune disease, end-stage renal failure, liver cirrhosis, and active cancer were excluded from our study. As HbA1c levels highly depend on the turnover of red blood cells, patients with iron, vitamin B12, and folate deficiency anemias were also excluded. Exclusion criteria also included erythropoietin therapy and hemolytic anemia for the previous reason. This way 112 diabetic, septic patients were included in our study (SIRS group) from whom 39 had HbA1c measured in the previous 30 days (SIRS 30 d subgroup) and 73 within 24 hours after their ED admission (SIRS 24 h subgroup). The past medical history (type of diabetes mellitus and date of diagnosis, hypertension, dyslipidemia, ischemic heart disease, previous myocardial infarction, percutaneous coronary intervention, coronary artery bypass grafting surgery, transient ischemic attack, stroke, peripheral arterial disease, chronic renal failure), antidiabetic therapy (metformin, sulfonylureas, dipeptidyl peptidase-4 inhibitors, other oral antidiabetic agents, insulin), laboratory results (arterial blood gas results, urea and electrolytes, glucose levels, liver function tests, pancreatic enzymes, C-reactive protein—CRP, procalcitonin—PCT, albumin, full blood count), HbA1c levels and time of measurement, SIRS and SOFA scores, microbiological results, type of infection, length of hospital stay, and mortality data of all patients were collected. Most laboratory parameters—with sometimes the exception of HbA1c—were measured upon arrival.

**Figure 1:**
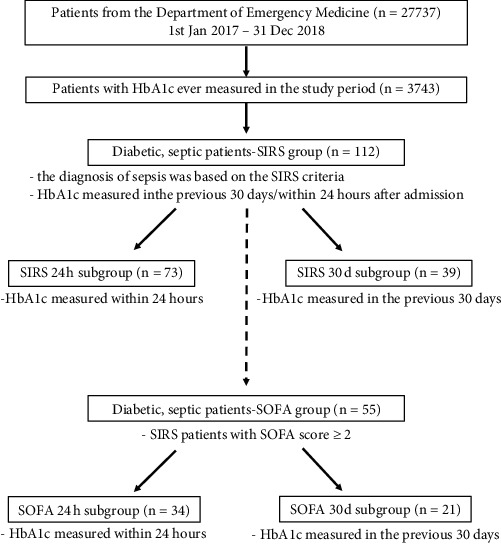
Flowchart showing the structure of the study, enrolment, and evaluation procedure, and how the patients were divided into groups and phases.

We later selected those patients from the SIRS group whose SOFA score was ≥2 (55 patients, SOFA group). Patients from the SOFA group were also divided into subgroups based on the time of measurement of HbA1c (patients with HbA1c measured in the previous 30 days—SOFA 30 d subgroup vs. patients with HbA1c measured within 24 hours after their ED admission—SOFA 24 h subgroup) (Figure 1).

The study conforms to the guiding principles of the Declaration of Helsinki, and our study subjects gave informed consent to a study that has been approved by the Institutional Committee on Human Research at our institution (Registration No.: DE RKEB/IKEB H.0172–2020).

### 2.2. Statistical Analyses

The STATISTICA 13.7 (TIBCO Inc., Tulsa, OK, USA) software was used for data analysis. The Kolmogorov-Smirnov test was used for testing the normality of data distribution. Results were either given as mean ± standard deviation in case of normal distribution or median (lower and upper quartile) in case of non-normal distribution, respectively. Since the distribution of some variables of interest became normal upon base-10 logarithm transformation, we used in the case of these variables the log values for correlation analyses. Pearson's univariate correlation was performed for finding significant relationships (in case of significant correlations, *p* was <0.05). Based on a recent review [38], variables that showed significance in the univariate analysis, as well as those that are clinically important, were included for multivariate analysis. Therefore, multiple regression analysis by the backward stepwise method was performed to determine independent predictor (*s*) of HbA1c. The model included age, gender, log10 length of hospital stay in survivors, insulin use, thrombocyte count, log10 bilirubin, white blood cell levels, and log10 fasting glucose. Variables that did not show correlations with HbA1c were excluded before analysis. Results were considered to be significant at the level of *p* < 0.05.

## 3. Results

### 3.1. Diabetic, Septic Patients with HbA1c Levels Measured within 24 hours after ED Admission

#### 3.1.1. SIRS 24 h Patients

**Figure 2:**
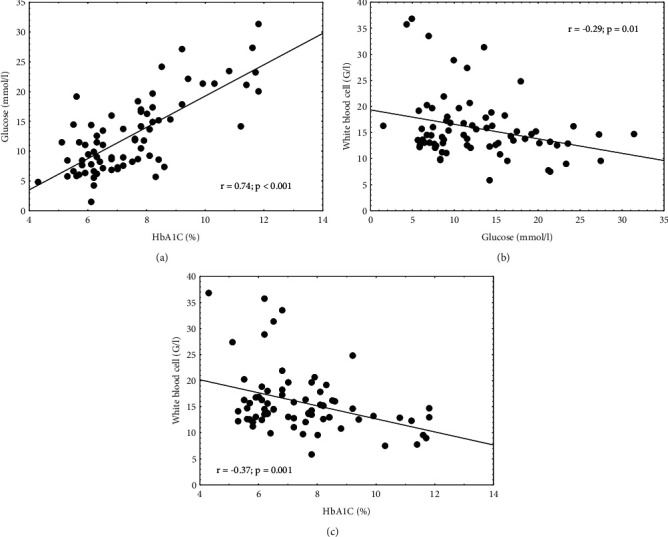
Correlations between glucose and HbA1C (%). (a) Glucose and white blood cell count. (b) White blood cell count and HbA1C (%) in diabetic, SIRS 24 h septic patients (*n* = 73).

SIRS 24 h patients were 72.8 ± 12.7 years old (73 patients: 47 females, 26 males). All our patients were type II diabetics. Anthropometric data, past medical history, antidiabetic therapy, laboratory parameters of patients as well as length of hospital stay in survivors were summarized in a table (Table 1). We analyzed the relationship between laboratory parameters and HbA1c as well as the correlation between length of hospital stay and HbA1c. Additionally, we examined the relationship between leukocyte count and glucose, platelet count and glucose, and length of hospital stay and glucose levels (Figure 1.). In these patients, there was a significant positive correlation between glucose and HbA1c levels (*p* < 0.001) (Figure 2(a)). We found significant negative correlations between white blood cell count and glucose (*p*=0.01) (Figure 2(b)), white blood cell count and HbA1c levels (*p*=0.001) (Figure 2(c)). The same correlations were observed in most cases even if patients were divided based on gender, antidiabetic therapy (oral antidiabetic agents vs. insulin therapy), age (<65 yrs vs. ≥65 yrs), and hospitalization in the previous 90 days (Table 2). We could not conclude anything regarding HbA1c and mortality due to the lack of data.

#### 3.1.2. SOFA 24 h Patients

**Figure 3:**
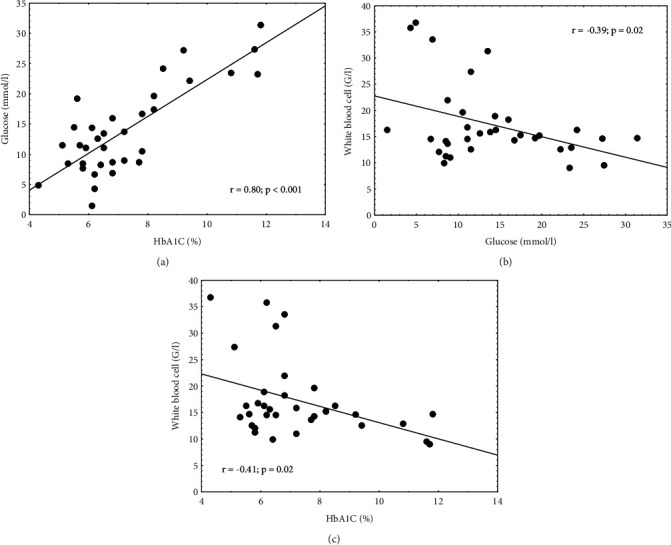
Correlations between glucose and HbA1C (%). (a) Glucose and white blood cell count. (b) White blood cell and HbA1C (%) in diabetic, SOFA 24 h septic patients (*n* = 34).

34 type II diabetic, septic patients were in the SOFA 24 h group (21 females, 13 males, age: 74 ± 12.3 years). Anthropometric data, past medical history, antidiabetic therapy, laboratory parameters of patients as well as length of hospital stay in survivors were summarized in a table (Table 1). We also analyzed the relationship between laboratory parameters and HbA1c as well as the correlation between length of hospital stay and HbA1c. Additionally, we examined the relationship between leukocyte count and glucose, platelet count and glucose, and length of hospital stay and glucose levels. There was a significant positive correlation between glucose and HbA1c levels in the SOFA 24 h group, similar to the one we found in SIRS 24 h patients (*p* < 0.001) (Figure 3(a)). We also found significant negative correlations between white blood cell count and glucose (*p*=0.02) (Figure 3(b)) and white blood cell count and HbA1c levels in SOFA 24 h patients (*p*=0.02) (Figure 3(c)). Additionally, there was a significant positive correlation between HbA1c levels and length of hospital stay in survivors (*p*=0.01) (data not shown). The previous correlations in the SOFA 24 h group were observed in most cases even if patients were divided based on gender, antidiabetic therapy (oral antidiabetic agents vs. insulin therapy), age (<65 yrs vs. ≥65 yrs), and hospitalization in the previous 90 days (Table 3). We could not conclude anything regarding HbA1c and mortality due to the lack of data.

### 3.2. Diabetic, Septic Patients with HbA1c Levels Measured in the Previous 30 Days before Their ED Admission

#### 3.2.1. SIRS 30 d and SOFA 30 d Patients

There were 39 diabetic, septic patients in the SIRS 30 d group. We studied the same correlations that were previously examined in the SIRS 24 h group. We did not find any significant correlation in this population even if we later selected and examined patients whose SOFA score was positive (≥2) (SOFA 30 d group, 21 patients) (data not shown). We could not conclude anything regarding HbA1c and mortality due to the lack of data.

### 3.3. Backward Stepwise Multiple Regression Analysis

We performed backward stepwise multiple regression analysis to determine independent predictors of HbA1C. The model included age, gender, log_10_ length of hospital stay in survivors, insulin use, thrombocyte count, log_10_ bilirubin, white blood cell levels, and log_10_ fasting glucose. Glucose levels (*β* = 0.324; *p*=0.02) and insulin use (*β* = 0.612; *p*=0.003) were significant independent predictors of HbA1c.

## 4. Discussion

As diabetes mellitus is a major risk factor of sepsis, we aimed to evaluate the possible effects of diabetes mellitus and poor glycemic control on the diagnosis of sepsis. This is the first study to evaluate the potential role of HbA1c in diabetic, septic patients. In SIRS 24 h patients, we found a significant positive correlation between glucose and HbA1c levels, while significant negative correlations were observed between white blood cell count and glucose, white blood cell count and HbA1c. Correlations were observed even if patients were divided based on gender, antidiabetic therapy (oral antidiabetic agents vs. insulin therapy), age (<65 yrs vs. ≥65 yrs), and hospitalization in the previous 90 days. One possible explanation behind the observed negative correlations between white blood cell count and glucose, white blood cell count and HbA1c is glucose toxicity, a phenomenon previously described in pancreatic beta cells [39–41]. According to previous studies, hyperglycemia in diabetic patients increases oxidative stress and induces glucose-induced apoptosis mainly in metabolically active cells (e.g., white blood cells in sepsis), resulting in cell death [42]. There are some diabetic, septic patients—in whom sepsis is diagnosed based on the SIRS criteria—whose white blood cell count is normal. These diabetic, septic patients with normal white blood cell counts (WBC count between 4–12 × 10^9^/l) have higher HbA1c levels. This observation is crucial as white blood cell count is an important part of the SIRS criteria (positive criterium: white blood cell count <4,000/mm³ or >12,000/mm³ or >10% bands). It may occur in diabetic, septic patients—in whom the diagnosis is based on the SIRS criteria—that white blood cell count is normal (between 4–12 × 10^9^/l), and there is only one other positive SIRS criterium (heart rate >90, temperature <36°C or >38°C, respiratory rate >20 or PaCO₂ <32 mmHg). According to the definition of sepsis—based on the SIRS criteria—these patients are not septic; however, the potential life-threatening immune processes might have already started. HbA1c—based on the negative correlation found between white blood cell count and HbA1c levels—can be a useful tool in finding these patients: in diabetic patients, normal white blood cell count (4–12 × 10^9^/l) with elevated HbA1c levels should be considered a positive SIRS criterium. Therefore, HbA1c—measured within 24 hours after admission (preferably upon arrival)—could turn out to be an efficient way to identify these diabetic, septic patients early and initiate sepsis treatment accordingly. Furthermore, large, multicentric studies are needed to confirm our hypothesis.

In the SOFA 24 h group, we found a significant positive correlation between glucose and HbA1c levels, significant negative correlations between white blood cell count and glucose, white blood cell count and HbA1c. We also found a significant positive correlation between length of hospital stay and HbA1c levels in survivors. A significant negative correlation was observed between white blood cell count and HbA1c in SOFA 24 h diabetic, septic patients similarly to the SIRS 24 h group. It must be noted that white blood cell count is not a SOFA criterium. Therefore, its correlation with HbA1c and consequently the possible early diagnostic potential of HbA1c is not that significant in SOFA patients. On the other hand—as there was a significant positive correlation between length of hospital and HbA1c levels in survivors—HbA1c may be a significant prognostic tool in diabetic, septic patients in whom the diagnosis is based on the SOFA criteria.

We did not find any significant correlation in SIRS 30 d patients. Previous studies found no significant difference between HbA1c levels measured on admission and 30 days earlier in critically ill patients [43]. Based on the same correlations, we found in SIRS 24 h patients should have been observed in SIRS 30 d patients. A possible explanation for this difference is that HbA1c in our study was measured within 30 days prior to these patients' ED admission, and not 30 days prior exactly, and HbA1c measured on admission correlates better with a glucose concentration of the previous weeks. We did not find any significant correlation in the SOFA 30 d group either.

### 4.1. Limitations

Some limitations must be noted. Despite our significant correlations, enrolment of a larger population might increase the statistical power. Additionally, HbA1c levels strongly depend on the turnover of red blood cells: slow turnover (e.g., in iron, vitamin B12, or folate deficiency anemias) often results in higher, whereas fast turnover (e.g., hemolytic anemia and erythropoietin therapy) leading to lower HbA1c levels [31–37, 44]. Therefore, all patients with the above-mentioned disorders have been excluded from the study. Furthermore, according to some studies, HbA1c levels vary among different racial and ethnic groups (higher levels in Afro-Americans and Asians). [37]. We enrolled only Caucasian patients.

Multiple regression analysis showed that insulin use and glucose are independent predictors of HbA1c. In our study, we aimed to identify the clinical parameters that can predict the severity of sepsis in diabetic patients using multiple regression analysis by the backward stepwise method. We believe that the statistical analyses that we used are appropriate and precise enough to identify the numerical contribution of individual factors' risk prediction. Moreover, these statistical methods are widely accepted in clinical studies. It must be noted that the use of ensemble modeling is another elegant approach to predictive analytics [45]. The proposed ensemble models are still a very good way to improve the current study and a huge opportunity to incorporate more data sources and get more accurate predictions regarding the hospitalization of patients. Furthermore, studies are needed with direct hospital information system (HIS) data access in order to make calculations on the massive dataset and with the participation of data mining experts in order to fully leverage the aforementioned methods.

## 5. Conclusions

Based on our results, we can conclude that even normal white blood cell count could be abnormal in diabetic, septic patients in whom the diagnosis is based on the SIRS criteria if an elevated HbA1c level is measured within 24 hours after admission (preferably upon arrival). Therefore, in these patients, normal white blood cell count (4–12 × 10^9^/l) with elevated HbA1c levels could be considered a positive SIRS criterium. Poor glycemic control—and hence elevated HbA1c—results in altered white blood cell response in case of an acute infection, and this has to be considered when diagnosing sepsis, especially when the SIRS criteria are used.

In diabetic, septic patients, in whom the diagnosis of sepsis is based on the SOFA score and HbA1c is measured within 24 hours after admission (preferably upon arrival), HbA1c could be an important prognostic tool as there is a significant positive correlation between HbA1c levels and length of hospital stay in survivors.

Based on our findings, HbA1c could turn on to be far more than a simple parameter of glycemic control, and it could also be a marker for the diagnosis of sepsis and may have values regarding hospital stay and mortality in septic diabetic patients.

Furthermore, multicenter studies focusing on the possible diagnostic and prognostic role of HbA1c in diabetic, septic patients are needed to verify our data.

## Figures and Tables

**Table 1:** Anthropometric data, antidiabetic therapy, and laboratory parameters of diabetic, SIRS 24 h, and SOFA 24 h septic patients.

Diabetic, septic patients		
Criteria	SIRS 24 h group	SOFA 24 h group
Number of patients (*n*)	73 (47f/26m)	34 (21f/13m)
Age (years)	72.8 ± 12.7	73.9 ± 12.3
Type 2 diabetes (*n*)	73	34
Comorbidities		
Hypertension (*n*; %)	67 (91.8)	31 (91.2)
Dyslipidemia (*n*; %)	31 (42.5)	12 (35.3)
IHD/AMI/PCI/CABG (*n*; %)	32 (43.8)	18 (52.9)
TIA/stroke (*n*; %)	15 (20.6)	6 (17.7)
Peripheral arterial disease (*n*; %)	42 (57.5)	18 (52.9)
Chronic kidney disease (*n*; %)	27 (37.0)	13 (38.2)
Antidiabetic medications		
Metformin (*n*; %)	30 (41.1)	11 (32.4)
Sulphonyl urea (*n*; %)	24 (32.9)	10 (29.4)
DPP4 (*n*; %)	4 (5.5)	0
Insulin (*n*; %)	18 (24.7)	11 (32.4)
Laboratory parameters		
Glucose (mmol/l)	11.5 (7.7–16.3)	12.05 (8.5–19.2)
HbA1C (%)	7.47 ± 1.8	7.26 ± 1.9
Urea (mmol/l)	8.4 (6–12.3)	9.85 (6.2–19.4)
Creatinine (*µ*mol/l)	99 (77–137)	118 (95–172)
Glomerular filtration rate (ml/min^*∗*^1.73 m2)	52 (38–75)	42 (27–61)
C-reactive protein (mg/l)	77 (21–151.5)	108 (21.3–246.3)
AST (U/L)	21 (17–33.5)	25 (16–42)
GGT (U/L)	35 (21–69)	40 (16–124)
ALT (U/L)	21 (14–32)	21 (14–37)
Total bilirubin (*µ*mol/l)	10 (6.5–17.4)	11.2 (6.3–33.6)
White blood cell count (G/L)	15.8 ± 6.1	17.3 ± 7.3
Red blood cell count (T/L)	4.3 ± 0.7	4.3 ± 0.7
Hemoglobin concentration (g/l)	129.8 ± 22.3	133.8 ± 19.4
Thrombocyte (G/L)	252.6 ± 76.9	251.0 ± 92.8
Length of hospital stay (day)	8 (6–11.5)	8 (7–11.5)

Data are presented as mean ± standard deviation or median (lower-upper quartile).

**Table 2:** Correlations between various laboratory parameters in subgroups of diabetic, SIRS 24 h septic patients.

	*n*	Glucose vs. HbA1c	Urea vs. HbA1c	Creatinine vs. HbA1c	CRP vs. HbA1c	Bilirubin vs. HbA1c	WBC vs. glucose	WBC vs. HbA1c	RBC vs. glucose	RBC vs. HbA1c	THR vs. HbA1c	LOS vs. HbA1c	LOS vs. glucose	LOS vs. WBC
All	73	*r* = 0.74 *p* < 0.001	Ns	ns	ns	ns	*r* = −0.29 *p*=0.01	*r* = −0.37 *p*=0.001	ns	ns	ns	ns	ns	ns
Males	26	*r* = 0.84 *p* < 0.001	Ns	ns	ns	*r* = −0.43 *p*=0.05	Ns	*r* = −0.48 *p*=0.01	ns	ns	*r* = −0.46 *p*=0.02	ns	ns	ns
Females	47	*r* = 0.70 *p* < 0.001	ns	ns	ns	ns	*r* = −0.28 *p*=0.05	*r* = −0.32 *p*=0.02	ns	ns	ns	ns	ns	ns
Noninsulin	53	*r* = 0.76 *p* < 0.001	ns	ns	ns	ns	*r* = −0.28 *p*=0.04	*r* = −0.35 *p*=0.01	ns	ns	ns	ns	ns	ns
Insulin	18	*r* = 0.69 *p*=0.001	ns	ns	ns	ns	Ns	*r* = −0.52 *p*=0.03	ns	ns	ns	ns	*r* = 0.57 *p*=0.02	ns
Under 65 yrs	17	*r* = 0.87 *p* < 0.001	ns	ns	ns	ns	Ns	*r* = −0.53 *p*=0.03	ns	ns	ns	ns	ns	ns
Over 65 yrs	56	*r* = 0.70 *p* < 0.001	ns	ns	ns	ns	*r* = −0.28 *p*=0.04	*r* = −0.33 *p*=0.01	ns	ns	ns	ns	ns	ns
Not stay within 90 days	66	*r* = 0.77 *p* < 0.001	ns	ns	ns	ns	*r* = −0.32 *p*=0.01	*r* = −0.38 *p*=0.001	ns	ns	ns	ns	ns	ns
Stay within 90 days	7	Ns	ns	ns	ns	ns	Ns	ns	ns	ns	ns	ns	ns	ns

CRP, C-reactive protein; HbA1c, hemoglobin A1c; LOS, length of stay in survivors; RBC, red blood cell; THR, thrombocyte; WBC, white blood cell.

**Table 3:** Correlations between various laboratory parameters in subgroups of diabetic, SOFA 24 h septic patients.

	*N*	Glucose vs. HbA1c	Urea vs. HbA1c	Creatinine vs. HbA1c	CRP vs. HbA1c	Bilirubin vs. HbA1c	WBC vs. glucose	WBC vs. HbA1c	RBC vs. glucose	RBC vs. HbA1c	THR vs. HbA1c	LOS vs. HbA1c	LOS vs. glucose	LOS vs. WBC
All	34	*r* = 0.80 *p*=0.001	ns	ns	ns	ns	*r* = −0.39 *p*=0.02	*r* = −0.41 *p*=0.02	ns	ns	ns	*r* = 0.45 *p*=0.01	*r* = 0.57 *p*=0.001	ns
Males	13	*r* = 0.95 *p*=0.001	ns	ns	ns	*r* = −0.59 *p*=0.05	Ns	ns	ns	ns	*r* = −0.62 *p*=0.02	ns	ns	ns
Females	21	*r* = 0.73 *p*=0.001	ns	ns	ns	ns	Ns	ns	ns	ns	ns	*r* = 0.58 *p*=0.01	*r* = 0.72 *p*=0.001	ns
Non-insulin	22	*r* = 0.82 *p*=0.001	ns	ns	ns	ns	*r* = −0.42 *p*=0.05	*r* = −0.44 *p*=0.03	ns	ns	ns	*r* = 0.56 *p*=0.05	*r* = 0.43 *p*=0.01	ns
Insulin	11	*r* = 0.79 *p*=0.005	ns	ns	ns	ns	Ns	ns	ns	ns	ns	*r* = 0.67 *p*=0.04	*r* = 0.65 *p*=0.05	ns
Under 65 yrs	7	*r* = 0.84 *p*=0.02	ns	ns	ns	ns	Ns	*r* = −0.80 *p*=0.03	ns	ns	ns	*r* = 0.82 *p*=0.02	*r* = 0.91 *p*=0.004	ns
Over 65 yrs	27	*r* = 0.80 *p*=0.001	ns	ns	ns	ns	Ns	ns	ns	ns	ns	*r* = 0.45 *p*=0.02	*r* = 0.55 *p*=0.004	ns
Not stay within 90 days	29	*r* = 0.80 *p*=0.001	ns	ns	ns	ns	*r* = −0.43 *p*=0.02	*r* = −0.41 *p*=0.03	ns	ns	ns	*r* = 0.62 *p*=0.001	*r* = 0.73 *p*=0.001	ns
Stay within 90 days	5	*r* = 0.96 *p*=0.01	ns	ns	ns	ns	Ns	ns	ns	ns	ns	ns	ns	ns

CRP, C-reactive protein; HbA1c, hemoglobin A1c; LOS, length of stay in survivors; RBC, red blood cell; THR, thrombocyte; WBC, white blood cell.

## Data Availability

All data generated or analyzed during this study are included in this published article. All data generated or analyzed during the current study are available from the corresponding author on reasonable request.
